# Correlation between the timing of endovascular coiling or microsurgical clipping and long-term outcomes of patients after aneurysmal subarachnoid haemorrhage

**DOI:** 10.1186/cc12281

**Published:** 2013-03-19

**Authors:** N Ojukwu, R Jain, S Wijayatilake, A Bellini, R Shetty, J Khan, G De la Cerda

**Affiliations:** 1 Queen's Hospital, London, UK

## Introduction

Aneurysmal subarachnoid haemorrhage (aSAH) is associated with a high morbidity and mortality. Although UK Anaesthesia Guidelines advocate early coiling or clipping of the aneurysm within the first 72 hours of admission for all grades of aSAH, the optimal timing of treatment and whether this is linked with better neurological long-term outcome are a subject of debate [1]. We aimed to investigate whether the timing of the occlusion of the aneurysm translates into better outcome.

## Methods

A retrospective analysis of prospective collected data in a tertiary neuroscience centre from January to September 2012. All patients were managed according to the local Guidelines for the management of aSAH. Outcome was assessed at 3 months using the extended Glasgow Outcome Scale (GOSE) defining good recovery as a GOSE ≥7 and poor outcome as GOSE ≤6.

## Results

**Figure 1:**
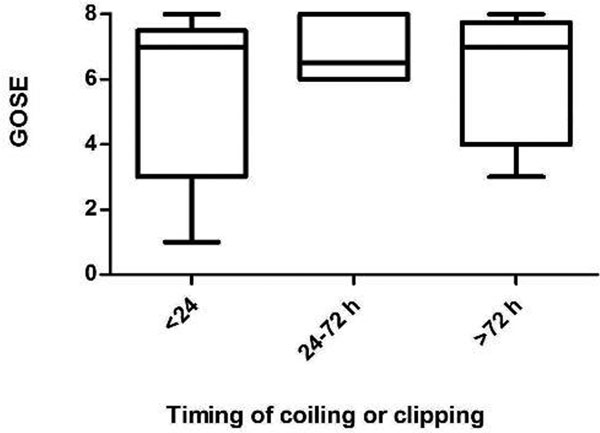
**Time of coiling/clipping versus GOSE boxplot**.

A total of 28 patients were included within the study period. Three patients were not expected to survive the first 24 hours and were not included in the study. Seventeen patients were classified as good grade aSAH (WFNS I to III) and eight as poor grade (IV toV). Twenty-two patients underwent successful coiling while the other three required clipping due to unsuccessful coiling. We did not find any correlation between the timing of coiling/clipping and the 3-month GOSE (Figure 1). A total 44% of the patients had a poor 3-month GOSE while 56% had a good long-term functional outcome. The overall mortality rate was 21%.

## Conclusion

Overall mortality in patients with aSAH is low when aneurysm is treated early post rupture of aneurysm. We did not find any correlation between the timing of occlusion of aneurysm and the 3-month functional outcome.
